# Supplementary material for: Muscular TOR knockdown and endurance exercise ameliorate high salt and age-related skeletal muscle degradation by activating the MTOR-mediated pathway
Source: PLoS One. 2025 Jan 22;20(1):e0311159. doi: 10.1371/journal.pone.0311159 (PMC11753686; doi:10.1371/journal.pone.0311159)
Supplement: Attachment — Submitted filename: Response to Reviewers.doc [file pone.0311159.s002.doc]

Reviewer #1: In this article, authors firstly conformed that the muscle TOR gene played an important role in endurance exercise against HSD-induced age-related skeletal muscle degeneration, as it determined the activity of the MTOR/SIR2/PGC-1α and MTOR/ATG2/PGC-1α pathways in skeletal muscle. The author provides a lot of data to support his conclusion. However, there were still some minor issues that need to be fixed to make the article better.
1. The shortened words should be standardized and uniform. For example, the article had TOR and MTOR, these two have the same meaning or different meaning.

The full names of TOR and MTOR have been added to distinguish them
2. The format and grammar of the article need to be carefully revised.

The format has been modified according to the requirements of the journal, and the syntax has been Revised. Please see the blue font in the body of the Revised Manuscript with Track Changes
3. The author should add some latest references for discussion and analysis.

It has been modified. For details, see the section highlighted in blue in Revised Manuscript with Track Changes.

Reviewer #2: The manuscript by Wang et al., studies the mechanism how exercise can ameliorate age-related skeletal muscle deterioration by high salt diet. This study can provide novel insight into how sarcopenia is caused. However there are several issues that need to be addressed:
Major issues
1. The reason why the authors used flies where TOR was either knocked-down or overexpressed for the experiments is unclear. The authors need to provide a better explanation. At the same time the authors need to add results of experiments obtained from wild type flies. Also, the authors need to prove that the RNAi for TOR indeed knocked-down the gene

TOR knockdown/overexpression flies were used to verify the role of TOR in this study. For example, from the differences in motor performance, antioxidant capacity and gene expression levels between the TOR overexpression group and the TOR normal expression group, we can see the role of TOR genes in these aspects..The construction of TOR overexpression/knockdown system was demonstrated by comparing the expression level of MTOR gene in flies with normal TOR expression group at three week-old without intervention.The results showed that there were significant differences in the expression level of MTOR gene between overexpression group/knockdown group and normal expression group(See text 3.1 for details).
2. MHC-GAL4 expression in not limited to skeletal muscles. It is expressed in other sites such as cardiac muscles. The authors need to address that in the text. They also need to use a more specific driver (refer to Zappia and Frolov, 2016) and repeat some of their more fundamental experiments.

The experimental methods and procedures related to skeletal muscle have been completed (related to overexpression/knockdown MTOR gene expression levels in Drosophila melanogaster over three weeks).We do not deny that MHC-Gal4 is expressed in other muscles. However, we only examined the skeletal muscle of fruit flies, and we plan to study the specific changes of TOR gene in other muscles in the future.
Minor Issues:
1. The manuscript has several grammatical errors.

The format has been modified according to the requirements of the journal, and the syntax has been Revised. Please see the blue font in the body of the Revised Manuscript with Track Changes
2. The authors need to provide the full forms of abbreviations while mentioning them for the first time.

It has been modified. For details, see the section highlighted in blue in Revised Manuscript with Track Changes.
3. The authors need to make sure that they consistently use the same nomenclature to refer to the same thing. For isntance they have both used weeks and weekends to denote age of flies.

It has been modified. For details, see the section highlighted in blue in Revised Manuscript with Track
4. The authors need to make sure that they used proper terminology throughout the paper consistent with previous publications. Also, they need to represent the genotypes of the flies correctly throughout the manuscript. Especially it will be helpful to have complete genotypes in the figures.

It has been modified. For details, see the section highlighted in blue in Revised Manuscript with Track
5. Information about rearing such as day-night cycle, temperature and humidity have not been mentioned.

For details, see Revised Manuscript with Track Changes 2.1.
